# Experiência Operatória e Desfechos Clínicos em Pacientes com Mixoma Cardíaco: Um Estudo Retrospectivo de Centro Único

**DOI:** 10.36660/abc.20250596

**Published:** 2026-03-03

**Authors:** Heleutério da Conceição Nicolau Madogolele, Manuela Cristina Ribeiro Dias Barroso, Hernán Patricio García Mejía, Cristhian Espinoza, Vinicius Machado Correia, Vagner Madrini, José Augusto Duncan, Ricardo Ribeiro Dias, Fábio Fernandes

**Affiliations:** 1 Hospital das Clínicas Faculdade de Medicina Universidade de São Paulo São Paulo SP Brasil Instituto do Coração do Hospital das Clínicas da Faculdade de Medicina da Universidade de São Paulo, São Paulo, SP – Brasil

**Keywords:** Mixoma, Cardiovascular Disease, Cirurgia, América Latina

## Abstract

**Fundamento:**

O mixoma cardíaco é o segundo tumor cardíaco primário mais comum; no entanto, séries contemporâneas realizadas em centro único que integrem estratégia operatória e desfechos clínicos ainda são limitadas.

**Objetivos:**

Descrever as abordagens operatórias e os desfechos clínicos de pacientes submetidos à ressecção cirúrgica de mixomas cardíacos em um único centro.

**Métodos:**

Realizamos uma revisão retrospectiva de 74 pacientes consecutivos submetidos à ressecção cirúrgica de mixomas cardíacos com confirmação histológica entre 2014 e 2024 em um centro terciário de referência no Brasil. Foram coletados dados clínicos, de imagem, cirúrgicos e de seguimento. O desfecho primário foi a incidência de eventos tromboembólicos. Foi adotado um nível de significância de 5% para todas as análises estatísticas.

**Resultados:**

A idade mediana foi de 60 anos (intervalo 52-66), e 68,9% dos pacientes eram do sexo feminino. O seguimento mediano foi de 60 meses (intervalo interquartil 40-79). Eventos tromboembólicos ocorreram em 14 pacientes (18,9%), incluindo acidente vascular encefálico (13,5%), infarto do miocárdio embólico (2,7%) e embolia pulmonar (2,7%). Na análise multivariada de Cox, o diabetes foi independentemente associado a menor risco de eventos tromboembólicos (HR 0,11; intervalo de confiança de 95% [IC 95%] 0,006-0,740; p < 0,05), enquanto a fibrilação atrial pós-operatória foi associada a maior risco tromboembólico (HR 6,74; IC 95% 1,490-30,510; p < 0,05). Recorrência tumoral ocorreu em 2,7% dos casos. Apenas um óbito foi registrado durante o seguimento.

**Conclusão:**

Os mixomas cardíacos estão associados a riscos tromboembólicos e hemodinâmicos significativos. A ressecção cirúrgica precoce proporciona excelentes desfechos em longo prazo e deve ser considerada mesmo em pacientes assintomáticos. Em nossa coorte, a morfologia tumoral apresentou uma tendência não significativa para maior risco de eventos tromboembólicos.

## Destaques/Pontos-chave

•Os mixomas cardíacos são os segundos tumores cardíacos primários mais comuns, e a ressecção cirúrgica permanece o tratamento de escolha.

•Neste estudo de centro único com 74 pacientes, os desfechos operatórios foram excelentes, com baixa taxa de recorrência (2,7%) e elevada sobrevida em longo prazo (98,6%).

•Eventos tromboembólicos ocorreram em 18,9% dos pacientes, com o diabetes associado a menor risco desses eventos.

•Esses achados sustentam a segurança e a eficácia em longo prazo da ressecção cirúrgica dos mixomas cardíacos, mesmo com uso conservador da reconstrução com *patch* interatrial.

## Graphical abstract

**Figura Central:**
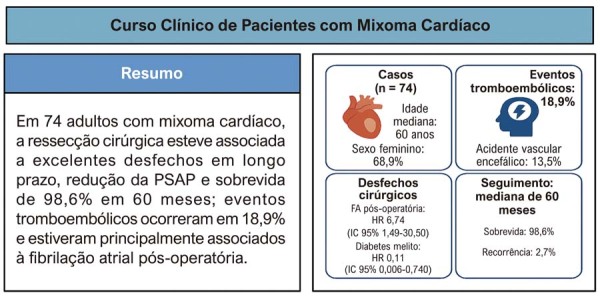
Experiência Operatória e Desfechos Clínicos em Pacientes com Mixoma Cardíaco: Um Estudo Retrospectivo de Centro Único

DM: diabetes melito; IC: intervalo de confiança; FA: fibrilação atrial; HR: razão de risco; PSAP: pressão sistólica da artéria pulmonar.

## Introdução

Os mixomas cardíacos são os segundos tumores cardíacos primários mais comuns, após os fibroelastomas papilares; no entanto, permanecem como as neoplasias cardíacas sintomáticas e tratadas cirurgicamente mais frequentes, correspondendo à maioria dos tumores cardíacos benignos ressecados na prática clínica.^1^ Apesar de sua histologia benigna, sua localização intracardíaca pode levar a morbidade significativa, incluindo obstrução, eventos embólicos e manifestações sistêmicas.^2^ Com uma incidência anual estimada de 0,5-1 caso por milhão, esses tumores raros ocorrem predominantemente em mulheres entre a terceira e a sexta décadas de vida e estão mais comumente localizados no átrio esquerdo (AE).^2 , 3^ A ressecção cirúrgica é o tratamento de escolha e está associada a desfechos favoráveis, embora a recorrência permaneça uma preocupação, particularmente em casos familiares.^4 , 5^

Na América Latina, especialmente no Brasil, os mixomas cardíacos correspondem a aproximadamente 70%-75% dos tumores cardíacos benignos relatados em séries cirúrgicas. No entanto, sua incidência populacional global permanece baixa, em torno de 0,5-1 caso por milhão por ano. Portanto, embora raros na população geral, representam a neoplasia cardíaca tratada cirurgicamente mais comum na região.^6^

Este estudo apresenta dados clínicos e cirúrgicos sobre mixomas cardíacos a partir de uma perspectiva latino-americana, onde as evidências disponíveis ainda são limitadas. Ao examinar padrões de recorrência e preditores de desfechos adversos, buscamos fornecer insights específicos da região que possam melhorar a avaliação de risco e orientar o manejo em longo prazo dos pacientes afetados.

## Métodos

### Desenho de estudo e aprovação ética

Este estudo retrospectivo analisou os prontuários médicos de pacientes diagnosticados com mixomas cardíacos e tratados em um hospital terciário no Brasil entre 2014 e 2024. O protocolo do estudo foi aprovado pelo Comitê de Ética em Pesquisa da mesma instituição (Protocolo SDC-COP 26501, aprovado em 12 de janeiro de 2025). Todos os dados foram totalmente anonimizados para garantir a confidencialidade dos pacientes, de acordo com os padrões éticos.

### População do estudo

Os participantes elegíveis foram pacientes adultos (≥ 18 anos) com diagnóstico confirmado de mixoma intracardíaco com base em exames de imagem, que foram submetidos à ressecção cirúrgica seguida de confirmação histopatológica e acompanhados nos ambulatórios da instituição por pelo menos 1 mês. Pacientes com idade > 45 anos ou com fatores de risco cardiovascular foram submetidos à avaliação pré-operatória institucional padrão, incluindo avaliação da perviedade das artérias coronárias para minimizar o risco de infarto do miocárdio perioperatório. A inclusão exigiu documentação médica completa, incluindo anotações clínicas e resultados de exames complementares, disponíveis no sistema institucional de prontuário eletrônico.

Os critérios de exclusão incluíram registros duplicados, documentação médica incompleta, cirurgia realizada em outra instituição, seguimento inferior a 30 dias e diagnóstico não confirmado de mixoma intracardíaco.

### Coleta de dados e variáveis

Os dados de seguimento foram obtidos exclusivamente do prontuário eletrônico institucional, sem contato direto com os pacientes. As seguintes variáveis foram coletadas: i) características demográficas e clínicas: idade, sexo, raça e comorbidades, como diabetes mellitus, hipertensão, histórico de tabagismo, dislipidemia e doença arterial periférica; ii) dados de apresentação e diagnóstico: sintomas iniciais e modalidade de imagem utilizada para o diagnóstico; iii) dados cirúrgicos: ano da intervenção cirúrgica, técnica cirúrgica e necessidade de procedimentos cardíacos adicionais; iv) parâmetros ecocardiográficos (registrados no pré e no pós-operatório por meio de protocolos padronizados realizados por ecocardiografistas certificados): fração de ejeção do ventrículo esquerdo (FEVE), diâmetro do AE, dimensões dos ventrículos esquerdo e direito (VD), função do VD e pressão sistólica da artéria pulmonar (PSAP); v) características tumorais: tamanho, morfologia, características da superfície, mobilidade, grau de obstrução e localização anatômica; vi) complicações e desfechos: eventos tromboembólicos, recorrência tumoral e eventos cardiovasculares adversos maiores.

Eventos cardiovasculares maiores durante o seguimento foram identificados por meio de revisão abrangente dos registros clínicos, resumos de alta hospitalar e, quando aplicável, certidões oficiais de óbito. Para garantir a precisão e consistência dos dados, todas as informações coletadas foram revisadas de forma independente por dois pesquisadores. As discrepâncias foram resolvidas por consenso ou avaliadas por um terceiro pesquisador.

Eventos tromboembólicos foram definidos como qualquer episódio tromboembólico clinicamente documentado confirmado por exames de imagem ou achados cirúrgicos, incluindo acidente vascular encefálico isquêmico, ataque isquêmico transitório, embolia sistêmica ou oclusão arterial periférica atribuível a fragmentos tumorais ou trombos. Os eventos também foram classificados de acordo com o momento de ocorrência em pré-operatórios, pós-operatórios precoces ou pós-operatórios tardios.

### Análise estatística

As variáveis contínuas foram expressas como mediana e intervalo interquartil, uma vez que nenhuma apresentou distribuição normal de acordo com o teste de Kolmogorov–Smirnov. As variáveis categóricas foram apresentadas como frequências absolutas e porcentagens.

As comparações entre grupos foram realizadas utilizando o teste de Mann–Whitney *U* para variáveis contínuas e o teste do qui-quadrado ou o teste exato de Fisher para variáveis categóricas, conforme apropriado. As comparações pareadas dos parâmetros ecocardiográficos pré e pós-operatórios foram avaliadas utilizando o teste de postos sinalizados de Wilcoxon. A sobrevida livre de eventos para desfechos tromboembólicos foi avaliada pelo método de Kaplan–Meier, com as curvas comparadas de forma descritiva.

Para identificar preditores de eventos tromboembólicos, foi inicialmente realizada análise de regressão de riscos proporcionais de Cox univariada. Variáveis com valor p < 0,10 foram incluídas no modelo multivariado para determinar associações independentes.

Todas as análises estatísticas foram realizadas utilizando o IBM SPSS Statistics for Windows, versão 27.0 (IBM Corp., Armonk, N.Y., EUA). Um valor p bicaudal < 0,05 foi considerado estatisticamente significativo.

## Resultados

### População do estudo e características basais

A amostra do estudo foi composta por 74 pacientes diagnosticados com mixoma cardíaco de acordo com os critérios de inclusão e exclusão predefinidos ( Diagrama 1 ). Os principais achados estão resumidos na Figura Central .

**Figure:**
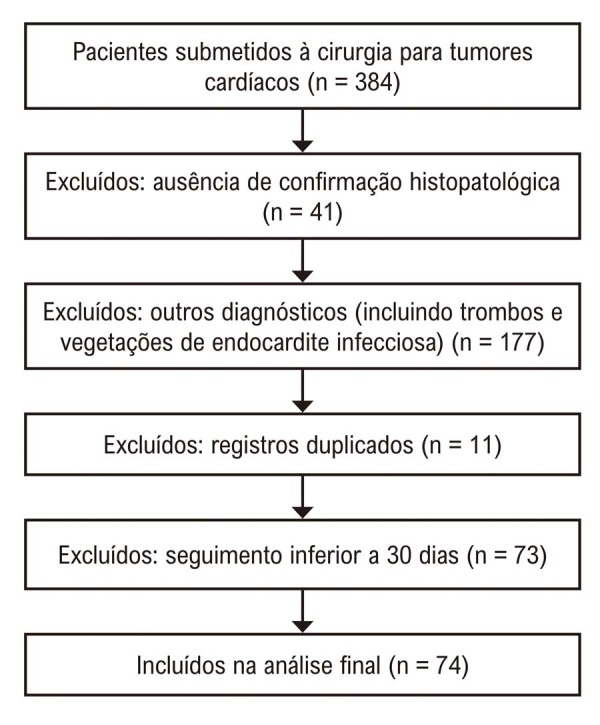
Diagrama 1 – Fluxograma ilustrando o processo de seleção dos pacientes incluídos no estudo. A maioria dos pacientes com seguimento inferior a 30 dias foi encaminhada de volta aos seus hospitais de origem após a primeira consulta pós-operatória.

As características demográficas e clínicas basais estão apresentadas na Tabela 1 . A coorte foi composta predominantemente por mulheres de meia-idade, com comorbidades cardiovasculares frequentes, particularmente hipertensão e dislipidemia. Entre os pacientes com neoplasias concomitantes, os tumores de tireoide e gastrointestinais foram os mais comuns. Dois pacientes apresentaram complexo de Carney confirmado, ambos com testes genéticos positivos identificando mutações no gene *PRKAR1A* .

**Tabela 1:** – Características basais por variáveis clínicas e epidemiológicas (n = 74)

Variáveis	Valor
**Dados clínicos**	
Idade (anos)	60 (52-66)
HAS, n (%)	56 (75,7)
Sexo feminino, n (%)	51 (68,9)
Raça branca, n (%)	61 (82,4)
Raça negra, n (%)	6 (8,1)
Diabetes melito, n (%)	24 (32,4)
Dislipidemia, n (%)	42 (56,8)
Histórico de tabagismo, n (%)	32 (43,2)
**Neoplasias concomitantes**	
Tireoide, n (%)	3 (4,1)
Mama, n (%)	1 (1,4)
Outras, n (%)	8 (10,8)
**Manifestações clínicas**	
Assintomático, n (%)	16 (21,8)
Dispneia, n (%)	33 (44,6)
Sintomas constitucionais, n (%)	13 (18,1)
Dor torácica, n (%)	6 (8,2)
Cardiopatia associada, n (%)	15 (20,3)
Forma obstrutiva, n (%)	27 (36,5)
**Modalidade diagnóstica pré-operatória**	
ETT, n (%)	57 (77,0)
ETE, n (%)	7 (9,5)
RMC, n (%)	10 (13,5)
**Diagnóstico histopatológico**	
Mixoma isolado, n (%)	69 (93,2)
Múltiplos mixomas, n (%)	5 (6,8)
**Procedimentos associados**	
Cirurgia da valva mitral, n (%)	14 (18,9)
Reconstrução com patch interatrial, n (%)	11 (14,9)
Cirurgia da valva tricúspide, n (%)	2 (2,7)
Cirurgia da valva aórtica, n (%)	1 (1,4)
**Seguimento**	
Duração do seguimento (meses), mediana [IIQ]	60 [40-79]

ETE: ecocardiografia transesofágica; ETT: ecocardiografia transtorácica; HAS: hipertensão arterial sistêmica; IIQ: intervalo interquartil; RMC: ressonância magnética cardíaca.

### Apresentação clínica

**Figura 1:**
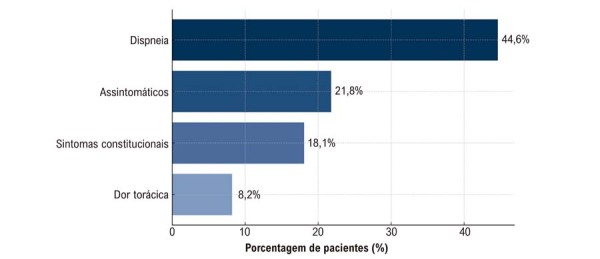
– Sintomas de apresentação em pacientes com mixoma cardíaco. Distribuição das principais manifestações clínicas na apresentação inicial.

Dispneia foi o sintoma inicial mais comum, refletindo a natureza obstrutiva dos mixomas cardíacos, particularmente daqueles localizados no AE. Notavelmente, 21,8% dos pacientes eram assintomáticos ( Figura 1 ), com tumores identificados incidentalmente em exames de imagem. Sintomas constitucionais, incluindo febre, perda de peso, anemia e astenia, foram documentados em 18,1% dos casos. Outros sintomas relatados incluíram dor torácica e palpitações, possivelmente relacionados a eventos tromboembólicos ou arritmias. Além disso, 36,5% dos tumores apresentaram padrão obstrutivo, contribuindo para sintomas aos esforços.

### Procedimentos cirúrgicos e estratégia operatória

**Figura 2:**
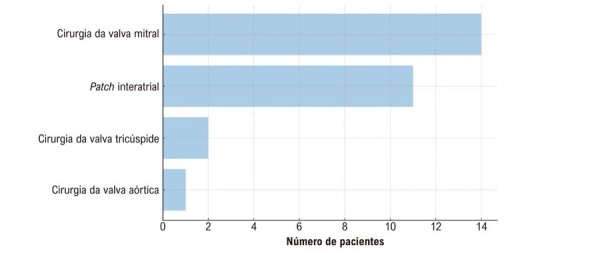
– Frequência de procedimentos cardíacos concomitantes realizados durante a ressecção do mixoma.

Entre os 74 pacientes, a cirurgia da valva mitral foi o procedimento associado mais frequente ( Figura 2 ). Em 12 casos, a intervenção foi necessária devido ao envolvimento direto do mixoma nas cúspides valvares, enquanto em dois pacientes o procedimento foi realizado de forma oportunística. Outros procedimentos associados incluíram correção com *patch* interatrial, cirurgia da valva tricúspide e cirurgia da valva aórtica.

Todos os pacientes foram submetidos à esternotomia mediana. Os mixomas do AE foram abordados por via transseptal através do átrio direito (AD), pela fossa oval quando havia suspeita de envolvimento septal ou implantação de base ampla, permitindo ressecção em bloco com um segmento do septo interatrial seguido de fechamento com *patch* , ou por atriotomia esquerda direta nos casos de lesões pediculadas ou localizadas na parede livre. Procedimentos valvares concomitantes foram realizados quando clinicamente indicados.

### Achados ecocardiográficos

No pré-operatório, a maioria dos pacientes apresentava fração de ejeção biventricular preservada e diâmetro diastólico do ventrículo esquerdo dentro da normalidade. Dilatação do AE foi observada em 41,9% dos casos, e PSAP elevada estava presente em 20,3% da coorte ( Tabela 2 ).

**Tabela 2:** – Achados ecocardiográficos pré e pós-operatórios e características dos mixomas

Variáveis	n (%) ou mediana (IIQ)
**Dados pré-operatórios**	
**FEVE**	
*** *** Preservada	69 (93,2)
*** *** Levemente reduzida	3 (4,1)
*** *** Gravemente reduzida	2 (2,7)
**Diâmetro diastólico do VE**	
Normal	66 (89,2)
Dilatado	8 (10,8)
**AE**	
Normal	43 (58,1)
Dilatado	31 (41,9)
**Função do VD**	
Normal	69 (93,2)
Disfunção leve	3 (4,1)
Disfunção moderada	2 (2,7)
**PSAP**	
Normal	59 (79,7)
Levemente aumentada	6 (8,1)
Gravemente aumentada	9 (12,2)
**Características do mixoma**	
**FEVE**	
Preservada	56 (78,9)
Levemente reduzida	4 (5,6)
Gravemente reduzida	11 (15,5)
**Diâmetro diastólico do VE**	
Normal	64 (86,5)
Dilatado	6 (8,1)
**AE**	
Normal	47 (63,5)
Dilatado	23 (31,1)
**Função do VD**	
Normal	64 (86,5)
Disfunção leve	6 (8,1)
Disfunção grave	1 (1,4)
**PASP**	
Normal	57 (82,6)
Levemente aumentada	9 (12,2)
Gravemente aumentada	3 (4,1)
**Características do mixoma**	
**Localização**	
AE	65 (87,8)
AD	4 (5,4)
Biatrial	2 (2,7)
**Morfologia**	
Pediculado	49 (68,1)
Séssil	23 (31,9)
**Superfície da lesão**	
Lisa	59 (79,7)
Vilosa	15 (20,3)
**Tamanho do tumor**	
Comprimento (mm)	45 (29-57)
Largura (mm)	35 (22-42)

AD: átrio direito; AE: átrio esquerdo; FEVE: fração de ejeção do ventrículo esquerdo (VE); IIQ: intervalo interquartil; PSAP: pressão sistólica da artéria pulmonar; VD: ventrículo direito.

No pós-operatório, a maioria dos pacientes manteve fração de ejeção biventricular preservada, embora a proporção com reduções graves da FEVE tenha aumentado em 12,8%. O tamanho normal do AE aumentou em 10,8%, e a normalização da PSAP foi observada em 6% dos pacientes ( Tabela 2 ).

**Figura 3:**
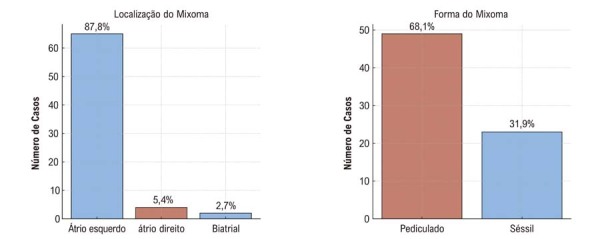
– Morfologia tumoral e principais localizações anatômicas.

**Figura 4:**
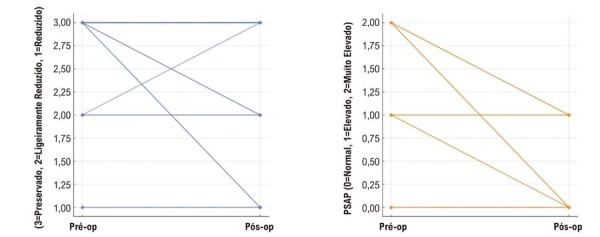
– Comparação dos parâmetros ecocardiográficos pré e pós-operatórios. Comparação da FEVE e da PSAP demonstrando redução significativa da PSAP no pós-operatório e queda transitória da FEVE. FEVE: fração de ejeção do ventrículo esquerdo; PSAP: pressão sistólica da artéria pulmonar.

Os mixomas estavam predominantemente localizados no AE ( Figura 3 ), geralmente com superfície lisa, e apresentaram dimensões medianas de 45 × 35 mm ( Tabela 2 ). O teste de postos sinalizados de Wilcoxon ( Figura 4 ) demonstrou diferenças estatisticamente significativas entre as medidas pré e pós-operatórias tanto para a FEVE quanto para a PSAP. Especificamente, ambos os parâmetros apresentaram reduções significativas, com a PSAP diminuindo principalmente em pacientes com mixomas obstrutivos.

### Complicações e desfechos clínicos

**Figura 5:**
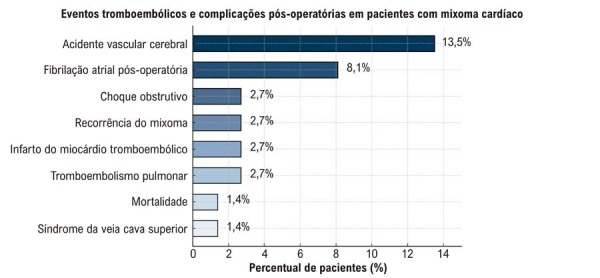
– Resumo dos eventos tromboembólicos e complicações pós-operatórias.

Eventos tromboembólicos foram observados em 18,9% dos pacientes, sendo o acidente vascular encefálico isquêmico a manifestação mais frequente. Todos os casos de AVC ocorreram em pacientes com fibrilação atrial pré-operatória. Os eventos ocorreram em 8,1% no pré-operatório e em 5,4% no pós-operatório ( Tabela 3 ). Complicações menos frequentes incluíram fibrilação atrial, choque obstrutivo e síndrome da veia cava superior ( Figura 5 ).

**Tabela 3:** – Desfechos de morbidade e mortalidade após ressecção de mixoma

Desfecho	n (%)
**Eventos tromboembólicos**	14 (18,9)
Embolia pulmonar	2 (2,7)
Acidente vascular encefálico	10 (13,5)
Infarto do miocárdio embólico	2 (2,7)
**Recorrência do mixoma**	2 (2,7)
**Fibrilação atrial pós-operatória**	6 (8,1)
**Choque obstrutivo**	2 (2,7)
**Síndrome da veia cava superior**	1 (1,4)
**Mortalidade**	1 (1,4)

**Figura 6:**
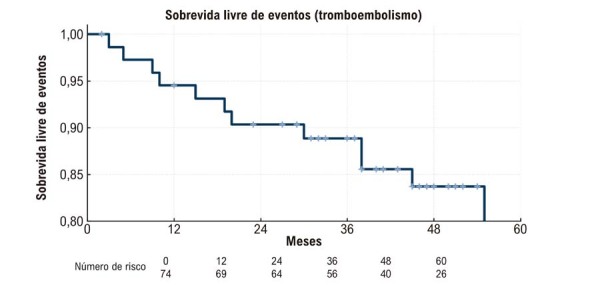
– Curva de Kaplan–Meier para sobrevida livre de eventos tromboembólicos. Sobrevida livre de eventos até 60 meses, mostrando que a maioria dos pacientes permaneceu livre de eventos tromboembólicos durante o seguimento.

A taxa de mortalidade foi baixa, com apenas um óbito registrado durante um período mediano de seguimento de 60 meses. A sobrevida livre de eventos foi adicionalmente ilustrada por meio de uma curva de Kaplan–Meier, que demonstrou um declínio progressivo na liberdade de eventos tromboembólicos ao longo do tempo ( Figura 6 ).

### Recorrência tumoral

A recorrência do mixoma foi incomum. Ambos os casos recorrentes envolveram múltiplos episódios (três recorrências cada) e estavam associados ao complexo de Carney. Um caso envolveu uma paciente do sexo feminino de 18 anos com três recorrências ventriculares esquerdas, e o outro um paciente do sexo masculino de 44 anos com envolvimento sequencial do AE, AD e, por fim, recorrência biatrial.

### Análise comparativa de acordo com eventos tromboembólicos

**Figura 7:**
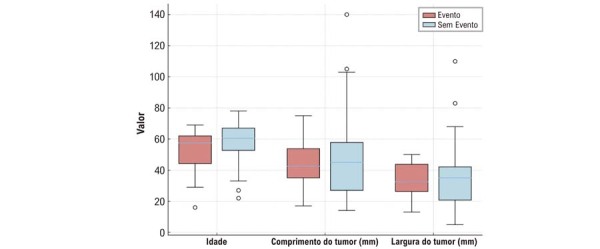
– Boxplots das variáveis clínicas de acordo com a ocorrência de eventos tromboembólicos. Distribuição da idade e do tamanho tumoral estratificada pela ocorrência de eventos tromboembólicos.

Pacientes que apresentaram eventos tromboembólicos (n = 14) foram comparados com aqueles que não apresentaram (n = 60) utilizando os testes do qui-quadrado ou exato de Fisher para variáveis categóricas e o teste de Mann–Whitney *U* para variáveis contínuas devido à distribuição não normal ( Figuras 7 e 8 ). A idade foi semelhante entre os grupos (mediana: 57,5 vs. 60,5 anos). Diabetes melito foi significativamente mais prevalente no grupo sem eventos (7,1% vs. 38,3%).

**Figura 8:**
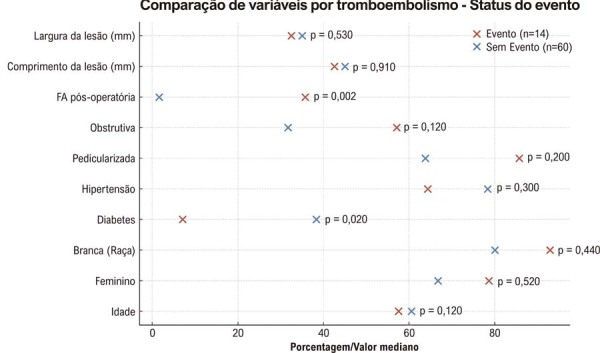
– Comparação das características clínicas e tumorais de acordo com a ocorrência de eventos tromboembólicos. FA: fibrilação atrial.

Outros fatores, incluindo tamanho tumoral, localização e parâmetros ecocardiográficos (FEVE, dilatação do AE e PSAP), não apresentaram diferenças estatisticamente significativas entre os grupos ( Tabela 4 ). Esses achados são consistentes com os resultados do modelo de riscos proporcionais de Cox apresentados nas Tabelas 5 e 6 , nos quais diabetes e fibrilação atrial emergiram como preditores independentes de eventos embólicos.

**Tabela 4:** – Comparação entre pacientes com e sem eventos tromboembólicos

Variável	Evento (n = 14)	Sem evento (n = 60)	Valor p
Idade, anos	57,5 (44,2-62,0)	60,5 (52,8-67,0)	0,120
Sexo feminino	11 (78,6%)	40 (66,7%)	0,520
Raça branca	13 (92,9%)	48 (80,0%)	0,440
Diabetes melito	1 (7,1%)	23 (38,3%)	0,020
HAS	9 (64,3%)	47 (78,3%)	0,300
Tumor pediculado	12 (85,7%)	37 (63,8%)	0,200
Padrão obstrutivo	8 (57,1%)	19 (31,7%)	0,120
FA pós-operatória	5 (35,7%)	1 (1,6%)	0,002
Comprimento tumoral (mm)	42,5 (35,0-53,8)	45,0 (27,0-57,8)	0,910
Largura tumoral (mm)	32,5 (26,2-43,8)	35,0 (20,8-42,0)	0,530

Os valores são apresentados como n (%) para variáveis categóricas e mediana [IIQ] para variáveis contínuas. Os valores de p foram calculados utilizando o teste exato de Fisher ou o teste de Mann–Whitney U, conforme apropriado. FA: fibrilação atrial; HAS: hipertensão arterial sistêmica; IIQ: intervalo interquartil.

**Tabela 5:** – Regressão de riscos proporcionais de Cox univariada para eventos tromboembólicos

Variável	HR	95% CI	Valor p
Idade (por ano)	0,96	0,92-1,00	0,06
Dislipidemia	1,02	0,31-3,30	0,97
HAS	2,00	0,57-7,03	0,27
FEVE reduzida no pré-operatório	2,22	0,62-7,86	0,21
Diâmetro diastólico do VE (dilatado)	0,33	0,06-1,60	0,17
PSAP aumentada	1,08	0,47-2,49	0,84
Mixoma móvel	1,87	0,52-6,71	0,33
Comprimento tumoral (mm)	0,99	0,96-1,02	0,67
Largura tumoral (mm)	1,00	0,96-1,03	0,98

IC: intervalo de confiança; FEVE: fração de ejeção do ventrículo esquerdo (VE); HAS: hipertensão arterial sistêmica; HR: razão de risco; PSAP: pressão sistólica da artéria pulmonar.

**Tabela 6:** – Regressão de riscos proporcionais de Cox multivariada para eventos tromboembólicos

Variável	HR	95% CI	Valor p
Diabetes melito	0,11	0,006-0,740	< 0,05
FA pós-operatória	6,74	1,49-30,51	< 0,05

IC: intervalo de confiança; FA: fibrilação atrial; HR: razão de risco.

### Preditores de eventos tromboembólicos

O modelo de regressão de Cox multivariado ( Tabela 6 ) mostrou que a dilatação do AE esteve associada a um menor risco, embora não significativo, de eventos tromboembólicos. Diabetes e fibrilação atrial estiveram significativamente associados ao risco de eventos tromboembólicos (HR = 0,11; IC 95%: 0,006-0,740; p < 0,05). Tumores móveis apresentaram tendência a maior risco de tromboembolismo, embora esse aumento não tenha sido estatisticamente significativo.

Análises univariadas ou multivariadas de recorrência do mixoma e mortalidade não foram realizadas devido ao pequeno número de eventos. Foi conduzida uma análise direcionada para explorar variáveis associadas a maior risco de eventos tromboembólicos. A análise univariada identificou o diabetes como um possível fator protetor, em contraste com seu papel estabelecido no aumento do risco tromboembólico na população geral. Esse achado sugere que o próprio mixoma pode ser o principal determinante do risco tromboembólico nessa população, reforçando a justificativa para a remoção cirúrgica precoce.

## Discussão

Este estudo reforça o papel crítico da intervenção cirúrgica precoce no manejo dos mixomas cardíacos e destaca sua associação com excelentes desfechos em longo prazo.^7^ Nesta coorte de 74 pacientes, predominantemente com tumores esporádicos, o seguimento mediano foi de 60 meses, durante os quais a sobrevida atingiu 98,6% e a recorrência tumoral permaneceu baixa, em 2,7%. Esses achados são consistentes com relatos prévios que demonstram a eficácia da ressecção imediata na redução da mortalidade e da recorrência, particularmente quando se alcança a excisão completa do tumor e de sua base de implantação.^8 , 9^

A menor frequência de sintomas constitucionais observada em nossa coorte contrasta com a maior prevalência relatada em outras séries. Essa diferença pode ser explicada pelos padrões de encaminhamento, uma vez que muitos pacientes foram avaliados em nosso centro em estágios mais avançados da doença, quando manifestações obstrutivas e embólicas podem predominar sobre sintomas sistêmicos.^10 - 12^

Todos os procedimentos em nossa coorte foram realizados por esternotomia mediana, que permanece a abordagem padrão em nossa instituição para garantir exposição adequada e excisão tumoral completa. Técnicas minimamente invasivas e robóticas, como aquelas que utilizam o sistema DaVinci Xi^®^, não estavam disponíveis durante o período do estudo. No entanto, essas abordagens têm sido associadas a tempos de recuperação mais curtos e melhor qualidade de vida pós-operatória em centros selecionados.^13 , 14^

O septo interatrial, particularmente a fossa oval, é reconhecido como o local de origem mais comum dos mixomas cardíacos. A ressecção em bloco com remoção de uma porção do septo interatrial seguida de reconstrução com *patch* é frequentemente recomendada para minimizar a recorrência. Em nossa coorte, apenas 14,9% dos pacientes foram submetidos à reconstrução com *patch* interatrial. Essa taxa relativamente baixa reflete uma estratégia cirúrgica individualizada e baseada na prática real em nosso centro terciário, onde as decisões operatórias foram guiadas pelo tamanho tumoral, morfologia da implantação e achados intraoperatórios.^15 - 18^

Em muitos casos, tumores pequenos e pediculados permitiram excisão completa com margem estreita e fechamento septal primário, particularmente em pacientes idosos ou de alto risco.^18 - 20^

A incidência de eventos tromboembólicos em nossa coorte (18,9%), principalmente acidente vascular encefálico isquêmico (13,5%), é comparável às taxas relatadas em estudos anteriores. Stefanou et al. (2018) descreveram uma taxa de AVC de 11% entre pacientes com mixomas cardíacos, enquanto Pinede et al. (2001) relataram eventos tromboembólicos em aproximadamente 30% dos casos. Importante destacar que nossa análise multivariada por regressão de Cox não identificou a localização tumoral nem a idade como preditores significativos de risco tromboembólico. A mobilidade tumoral apresentou tendência a aumento do risco, embora essa associação não tenha alcançado significância estatística, provavelmente devido ao número limitado de eventos. Essa observação é consistente com a metanálise de Liu et al. (2020), que destacou a ausência de preditores morfológicos consistentes entre os estudos.^3 , 21 , 22^

A fibrilação atrial pós-operatória emergiu como preditor independente de eventos tromboembólicos em nossa coorte, com aumento superior a seis vezes no risco. Esse achado reforça a associação bem estabelecida entre fibrilação atrial de início recente e maior risco embólico após cirurgia cardíaca e ressalta a importância do monitoramento rigoroso do ritmo e da anticoagulação oportuna nessa população.^23 , 24^

No modelo multivariado, o diabetes mellitus esteve associado a menor risco de eventos tromboembólicos. Essa observação não tem sido consistentemente relatada em estudos prévios e deve ser interpretada com cautela. É provável que tenha caráter exploratório e potencialmente confuso, em vez de indicar um verdadeiro efeito biológico protetor.^25 - 27^ Possíveis explicações incluem monitoramento clínico mais próximo, uso mais frequente de terapia antitrombótica ou estatinas, ou diferenças não mensuradas no manejo do risco cardiovascular entre pacientes diabéticos. Devido ao pequeno número de eventos e ao delineamento retrospectivo, esse achado deve ser considerado gerador de hipóteses e requer confirmação em estudos prospectivos maiores.

A coorte demonstrou redução significativa da PSAP no pós-operatório (Wilcoxon Z = −2,070; p = 0,038), consistente com o alívio da obstrução ao fluxo de entrada no AE, particularmente em pacientes com mixomas obstrutivos.

Por outro lado, a FEVE diminuiu no período pós-operatório precoce (FEVE preservada 93,2% no pré-operatório vs. 78,9% no pós-operatório; redução grave 2,7% vs. 15,5%; Wilcoxon Z = 3,052; p = 0,002). Esse padrão provavelmente reflete disfunção ventricular transitória perioperatória, e não deterioração sustentada da função sistólica, especialmente considerando que os ecocardiogramas pós-operatórios foram realizados dentro de 30 dias após a cirurgia. Esses achados sugerem que a cirurgia alivia prontamente a sobrecarga hemodinâmica, enquanto a recuperação da função ventricular pode ocorrer de forma mais gradual e requer acompanhamento ecocardiográfico longitudinal.

Mikus et al. (2025) relataram FEVE preservada no período pós-operatório, indicando que as alterações observadas em nossa coorte podem representar modificações precoces e transitórias, e não comprometimento em longo prazo.^14^

Por fim, a taxa de sobrevida observada em nosso estudo (98,6% ao longo de um seguimento mediano de 60 meses) é favorável quando comparada a séries cirúrgicas globais, incluindo a sobrevida em longo prazo de 93%–97% relatada por Jiang et al. (2019) e Perek et al. (2011). Esses resultados reforçam a segurança e a eficácia em longo prazo do tratamento cirúrgico dos mixomas cardíacos quando realizado em centros especializados.^7 , 9 , 28^

### Limitações do estudo

O delineamento retrospectivo, de centro único, e o tamanho relativamente pequeno da amostra podem limitar a generalização desses achados. Além disso, devido ao longo período de coleta de dados inerente a estudos retrospectivos, algumas variáveis potencialmente relevantes, como morfologia tumoral e características da superfície, que podem estar associadas à mobilidade e ao risco tromboembólico, não foram consistentemente documentadas.

Além disso, a redução pós-operatória observada na FEVE pode refletir parcialmente lesão miocárdica perioperatória ou infarto, o que não foi sistematicamente avaliado ou registrado em nossa coorte, limitando a interpretação desse achado.

## Conclusão

Nosso estudo reforça que o mixoma cardíaco, quando tratado precocemente, está associado a excelente prognóstico. Ao mesmo tempo, destaca o risco tromboembólico significativo relacionado a essa condição, que parece ser amplamente independente da morfologia tumoral. Esses achados apoiam a ressecção cirúrgica precoce, mesmo em pacientes assintomáticos e naqueles com lesões não obstrutivas. O acompanhamento contínuo permanece essencial para monitorar complicações tardias e possível recorrência.
