# Supplementary material for: Experiência Operatória e Desfechos Clínicos em Pacientes com Mixoma Cardíaco: Um Estudo Retrospectivo de Centro Único
Source: Arq Bras Cardiol. 2026 Mar 3;123(2):e20250596. [Article in Portuguese] doi: 10.36660/abc.20250596 (PMC13128183; doi:10.36660/abc.20250596)
Supplement: Translation

# Translation: Operative Experience and Clinical Outcomes in Cardiac Myxoma Patients: A Retrospective Single-Center Study

**DOI:** 10.36660/abc.20250596i

**Authors:** Heleutério da Conceição Nicolau Madogolele, Manuela Cristina Ribeiro Dias Barroso, Hernán Patricio García Mejía, Cristhian Espinoza, Vinicius Machado Correia, Vagner Madrini, José Augusto Duncan, Ricardo Ribeiro Dias, Fábio Fernandes

**Affiliations:** 1 Brazil Instituto do Coração do Hospital das Clínicas da Faculdade de Medicina da Universidade de São Paulo, São Paulo, SP – Brazil

**Keywords:** Mixoma, Doenças Cardiovasculares, Surgery, Latin America

## Abstract

**Background:**

Cardiac myxoma is the second most common primary cardiac tumor; however, contemporary single-center series integrating operative strategy with clinical outcomes remain limited.

**Objectives:**

To describe the operative approaches and clinical outcomes of patients undergoing surgical resection of cardiac myxomas at a single center.

**Methods:**

We conducted a retrospective review of 74 consecutive patients who underwent surgical resection of histologically confirmed cardiac myxomas between 2014 and 2024 at a tertiary referral center in Brazil. Clinical, imaging, surgical, and follow-up data were collected. The primary outcome was the incidence of thromboembolic events. A significance level of 5% was adopted for all statistical analyses.

**Results:**

The median age was 60 years (range 52-66), and 68.9% of patients were female. Median follow-up was 60 months (interquartile range 40-79). Thromboembolic events occurred in 14 patients (18.9%), including stroke (13.5%), embolic myocardial infarction (2.7%), and pulmonary embolism (2.7%). In multivariable Cox analysis, diabetes was independently associated with a lower hazard of thromboembolic events (HR 0.11; 95% CI 0.006-0.740; p < 0.05), whereas postoperative atrial fibrillation was associated with a higher thromboembolic risk (HR 6.74; 95% CI 1.490-30.510; p < 0.05). Tumor recurrence occurred in 2.7% of cases. Only one death was recorded during follow-up.

**Conclusion:**

Cardiac myxomas are associated with significant thromboembolic and hemodynamic risks. Early surgical resection provides excellent long-term outcomes and should be considered even in asymptomatic patients. In our cohort, tumor morphology showed a non-significant trend toward a higher risk of thromboembolic events.

## Highlights/Key points

•Cardiac myxomas are the second most common primary cardiac tumors, and surgical resection remains the treatment of choice.

•In this single-center study of 74 patients, operative outcomes were excellent, with a low recurrence rate (2.7%) and high long-term survival (98.6%).

•Thromboembolic events occurred in 18.9% of patients, with diabetes associated with a lower hazard of these events.

•These findings support the safety and long-term effectiveness of surgical resection for cardiac myxomas, even with conservative use of interatrial patch reconstruction.

## Introduction

Cardiac myxomas are the second most common primary cardiac tumors, after papillary fibroelastomas; however, they remain the most frequent symptomatic and surgically treated cardiac neoplasms, accounting for the majority of resected benign cardiac tumors in clinical practice.^1^Despite their benign histology, their intracardiac location can lead to substantial morbidity, including obstruction, embolic events, and systemic manifestations.^2^With an estimated annual incidence of 0.5-1 case per million, these rare tumors occur predominantly in women between the third and sixth decades of life and are most commonly located in the left atrium (LA).^2 , 3^Surgical resection is the treatment of choice and is associated with favorable outcomes, although recurrence remains a concern, particularly in familial cases.^4 , 5^

In Latin America, especially in Brazil, cardiac myxomas account for approximately 70%-75% of benign cardiac tumors reported in surgical series. However, their overall population incidence remains low, at approximately 0.5-1 case per million per year. Therefore, although rare in the general population, they represent the most common surgically treated cardiac neoplasm in the region.^6^

This study presents clinical and surgical data on cardiac myxomas from a Latin American perspective, where available evidence remains limited. By examining recurrence patterns and predictors of adverse outcomes, we aim to provide region-specific insights that may improve risk assessment and inform the long-term management of affected patients.

## Methods

### Study design and ethical approval

This retrospective study analyzed the medical records of patients diagnosed with cardiac myxomas and treated at a tertiary hospital in Brazil between 2014 and 2024. The study protocol was approved by the Institutional Review Board of the same institution (Protocol SDC-COP 26501, approved on January 12, 2025). All data were fully anonymized to ensure patient confidentiality in accordance with ethical standards.

### Study population

Eligible participants were adult patients (≥ 18 years) with a confirmed diagnosis of intracardiac myxoma based on imaging studies, who underwent surgical resection followed by histopathological confirmation, and who were followed in the institution’s outpatient clinics for at least 1 month. Patients aged > 45 years or those with cardiovascular risk factors underwent standard preoperative institutional evaluation, including assessment of coronary artery patency to minimize the risk of perioperative myocardial infarction. Inclusion required complete medical documentation, including clinical notes and complementary test results, available in the institutional electronic medical record system.

Exclusion criteria comprised duplicate records, incomplete medical documentation, surgery performed at another institution, follow-up shorter than 30 days, and unconfirmed diagnosis of intracardiac myxoma.

### Data collection and variables

Follow-up data were obtained exclusively from the institutional electronic medical record, with no direct patient contact. The following variables were collected: i) demographic and clinical characteristics: age, sex, race, and comorbidities, such as diabetes mellitus, hypertension, smoking history, dyslipidemia, and peripheral arterial disease; ii) presentation and diagnostic data: initial symptoms and the imaging modality used for diagnosis; iii) surgical data: year of surgical intervention, surgical technique, and the need for additional cardiac procedures; iv) echocardiographic parameters (recorded preoperatively and postoperatively using standardized protocols performed by certified echocardiographers): left ventricular ejection fraction (LVEF), LA diameter, left and right ventricular (RV) dimensions, RV function, and pulmonary artery systolic pressure (PASP); v) tumor characteristics: size, morphology, surface features, mobility, degree of obstruction, and anatomical location; vi) complications and outcomes: thromboembolic events, tumor recurrence, and major adverse cardiovascular events.

Major cardiovascular events during follow-up were identified through a comprehensive review of clinical records, hospital discharge summaries, and, when applicable, official death certificates. To ensure data accuracy and consistency, all collected information was independently reviewed by two researchers. Discrepancies were resolved by consensus or adjudicated by a third researcher.

Thromboembolic events were defined as any clinically documented thromboembolic episode confirmed by imaging or surgical findings, including ischemic stroke, transient ischemic attack, systemic embolism, or peripheral arterial occlusion attributable to tumor fragments or thrombus. Events were further classified according to timing as preoperative, early postoperative, or late postoperative.

### Statistical analysis

Continuous variables were expressed as median and interquartile range, as none followed a normal distribution according to the Kolmogorov–Smirnov test. Categorical variables were summarized as absolute frequencies and percentages.

Comparisons between groups were performed using the Mann–Whitney *U* test for continuous variables and the chi-square test or Fisher’s exact test for categorical variables, as appropriate. Paired comparisons of preoperative and postoperative echocardiographic parameters were evaluated using the Wilcoxon signed-rank test. Event-free survival for thromboembolic outcomes was assessed using the Kaplan–Meier method, with curves compared descriptively.

To identify predictors of thromboembolic events, univariate Cox proportional hazards regression analysis was initially performed. Variables with a p-value < 0.10 were entered into the multivariable model to determine independent associations.

All statistical analyses were conducted using IBM SPSS Statistics for Windows, version 27.0 (IBM Corp., Armonk, N.Y., USA). A two-tailed p-value < 0.05 was considered statistically significant.

## Results

### Study population and baseline characteristics

The study sample consisted of 74 patients diagnosed with cardiac myxoma according to the predefined inclusion and exclusion criteria ( Diagram 1 ). The main findings are summarized in Central Illustration .

**Diagram 1:** – Flowchart illustrating the selection process of patients included in the study. Most patients with follow-up shorter than 30 days were referred back to their originating hospitals after the first postoperative consultation.

Baseline demographic and clinical characteristics are presented in Table 1 . The cohort was composed predominantly of middle-aged women, with frequent cardiovascular comorbidities, particularly hypertension and dyslipidemia. Among patients with concomitant neoplasms, thyroid and gastrointestinal tumors were the most common. Two patients had confirmed Carney complex, both with positive genetic testing identifying *PRKAR1A* gene mutations.

**Table 1:** – Baseline characteristics by clinical and epidemiological variables (n = 74)

Variables	Value
**Clinical data**	
Age (years)	60 (52-66)
Hypertension, n (%)	56 (75.7)
Female sex, n (%)	51 (68.9)
White race, n (%)	61 (82.4)
Black race, n (%)	6 (8.1)
Diabetes mellitus, n (%)	24 (32.4)
Dyslipidemia, n (%)	42 (56.8)
Smoking history, n (%)	32 (43.2)
**Concomitant neoplasms**	
Thyroid, n (%)	3 (4.1)
Breast, n (%)	1 (1.4)
Other, n (%)	8 (10.8)
**Clinical manifestations**	
Asymptomatic, n (%)	16 (21.8)
Dyspnea, n (%)	33 (44.6)
Constitutional symptoms, n (%)	13 (18.1)
Chest pain, n (%)	6 (8.2)
Associated heart disease, n (%)	15 (20.3)
Obstructive form, n (%)	27 (36.5)
**Preoperative diagnostic modality**	
TTE, n (%)	57 (77.0)
TEE, n (%)	7 (9.5)
CMR, n (%)	10 (13.5)
**Histopathological diagnosis**	
Isolated myxoma, n (%)	69 (93.2)
Multiple myxomas, n (%)	5 (6.8)
**Associated procedures**	
Mitral valve surgery, n (%)	14 (18.9)
Interatrial patch reconstruction, n (%)	11 (14.9)
Tricuspid valve surgery, n (%)	2 (2.7)
Aortic valve surgery, n (%)	1 (1.4)
**Follow-up**	
Follow-up duration (months), median [IQR]	60 [40-79]

CMR: cardiac magnetic resonance; IQR: interquartile range; TEE: transesophageal echocardiography; TTE: transthoracic echocardiography.

### Clinical presentation

Dyspnea was the most common presenting symptom, reflecting the obstructive nature of cardiac myxomas, particularly those located in the LA. Notably, 21.8% of patients were asymptomatic ( Figure 1 ), with tumors identified incidentally on imaging. Constitutional symptoms, including fever, weight loss, anemia, and asthenia, were documented in 18.1% of cases. Additional reported symptoms included chest pain and palpitations, possibly related to thromboembolic events or arrhythmias. Furthermore, 36.5% of tumors exhibited an obstructive pattern, contributing to exertional symptoms.

**Figure 1:** – Presenting symptoms in patients with cardiac myxoma. Distribution of the main clinical manifestations at initial presentation.

### Surgical procedures and operative strategy

Among the 74 patients, mitral valve surgery was the most frequent associated procedure ( Figure 2 ). In 12 cases, intervention was required due to direct myxoma involvement of the valve leaflets, while in two patients the procedure was performed opportunistically. Other associated procedures included interatrial patch repair, tricuspid valve surgery, and aortic valve surgery.

**Figure 2:** – Frequency of concomitant cardiac procedures performed during myxoma resection.

All patients underwent median sternotomy. LA myxomas were approached either through a right atrial (RA) transseptal route via the fossa ovalis when septal involvement or broad-based implantation was suspected, allowing en bloc excision with a cuff of interatrial septum followed by patch closure, or through direct left atriotomy for pedunculated or free-wall lesions. Concomitant valve procedures were performed when clinically indicated.

### Echocardiographic findings

Preoperatively, most patients had preserved biventricular ejection fraction and normal left ventricular diastolic diameter. LA dilation was observed in 41.9% of cases, and elevated PASP was present in 20.3% of the cohort ( Table 2 ).

**Table 2:** – Preoperative and postoperative echocardiographic findings and myxoma characteristics

Variables	(%) or median (IQR)
**Preoperative data**	
**LVEF**	
Preserved	69 (93.2)
Mildly reduced	3 (4.1)
Severely reduced	2 (2.7)
**LV diastolic diameter**	
Normal	66 (89.2)
Dilated	8 (10.8)
**LA**	
Normal	43 (58.1)
Dilated	31 (41.9)
**RV function**	
Normal	69 (93.2)
Mild dysfunction	3 (4.1)
Moderate dysfunction	2 (2.7)
**PASP**	
Normal	59 (79.7)
Mildly increased	6 (8.1)
Severely increased	9 (12.2)
**Postoperative data**	
**LVEF**	
Preserved	56 (78.9)
Mildly reduced	4 (5.6)
Severely reduced	11 (15.5)
**LV diastolic diameter**	
Normal	64 (86.5)
Dilated	6 (8.1)
**LA**	
Normal	47 (63.5)
Dilated	23 (31.1)
**RV function**	
Normal	64 (86.5)
Mild dysfunction	6 (8.1)
Severe dysfunction	1 (1.4)
**PASP**	
Normal	57 (82.6)
Mildly increased	9 (12.2)
Severely increased	3 (4.1)
**Myxoma characteristics**	
**Location**	
LA	65 (87.8)
RA	4 (5.4)
Biatrial	2 (2.7)
**Morphology**	
Pedunculated	49 (68.1)
Sessile	23 (31.9)
**Lesion surface**	
Smooth	59 (79.7)
Villous	15 (20.3)
**Tumor size**	
Length (mm)	45 (29-57)
Width (mm)	35 (22-42)

IQR: interquartile range; LA: left atrium; LV: left ventricle; LVEF: LV ejection fraction; PASP: pulmonary artery systolic pressure; RA: right atrium; RV: right ventricle ***.***

Postoperatively, most patients maintained preserved biventricular ejection fraction, although the proportion with severe reductions in LVEF increased by 12.8%. Normal LA size increased by 10.8%, and normalization of PASP was observed in 6% of patients ( Table 2 ).

Myxomas were predominantly located in the LA ( Figure 3 ), generally with a smooth surface, and had median dimensions of 45 × 35 mm ( Table 2 ). A Wilcoxon signed-rank test ( Figure 4 ) demonstrated statistically significant differences between preoperative and postoperative measurements for both LVEF and PASP. Specifically, both parameters showed significant reductions, with PASP decreasing primarily in patients with obstructive myxomas.

**Figure 3:** – Tumor morphology and primary anatomical locations.

**Figure 4:** – Comparison of preoperative and postoperative echocardiographic parameters. Comparison of LVEF and PASP demonstrating a significant postoperative reduction in PASP and a transient decline in LVEF. LVEF: left ventricular ejection fraction. PASP: pulmonary artery systolic pressure.

### Complications and clinical outcomes

Thromboembolic events were observed in 18.9% of patients, with ischemic stroke as the most frequent manifestation. All stroke cases occurred in patients with preoperative atrial fibrillation. Events occurred in 8.1% preoperatively and in 5.4% postoperatively ( Table 3 ). Less frequent complications included atrial fibrillation, obstructive shock, and superior vena cava syndrome ( Figure 5 ).

**Table 3:** – Morbidity and mortality outcomes after myxoma resection

Outcome	n (%)
**Thromboembolic events**	14 (18.9)
Pulmonary embolism	2 (2.7)
Stroke	10 (13.5)
Embolic myocardial infarction	2 (2.7)
**Myxoma recurrence**	2 (2.7)
**Postoperative atrial fibrillation**	6 (8.1)
**Obstructive shock**	2 (2.7)
**Superior vena cava syndrome**	1 (1.4)
**Mortality**	1 (1.4)

**Figure 5:** – Summary of thromboembolic events and postoperative complications.

The mortality rate was low, with only one death recorded during a median follow-up period of 60 months. Event-free survival was further illustrated using a Kaplan–Meier curve, which demonstrated a progressive decline in freedom from thromboembolic events over time ( Figure 6 ).

**Figure 6:** – Kaplan–Meier curve for thromboembolic event-free survival. Event-free survival up to 60 months, showing that most patients remained free from thromboembolic events during follow-up.

### Tumor recurrence

Myxoma recurrence was uncommon. Both recurrent cases involved multiple episodes (three recurrences each) and were associated with Carney complex. One case involved an 18-year-old female with three left ventricular recurrences, and the other a 44-year-old male with sequential involvement of the LA, RA, and eventually a biatrial recurrence.

### Comparative analysis according to thromboembolic events

Patients who experienced thromboembolic events (n = 14) were compared with those who did not (n = 60) using chi-square or Fisher’s exact tests for categorical variables and the Mann–Whitney *U* test for continuous variables due to non-normal distribution ( Figures 7 and 8 ). Age was similar between groups (median: 57.5 vs. 60.5 years). Diabetes mellitus was significantly more prevalent in the event-free group (7.1% vs. 38.3%).

**Figure 7:** – Boxplots of clinical variables according to thromboembolic event status. Distribution of age and tumor size stratified by the occurrence of thromboembolic events.

**Figure 8:** – Comparison of clinical and tumor characteristics by thromboembolic event status. AF: atrial fibrillation.

Other factors, including tumor size, location, and echocardiographic parameters (LVEF, LA dilation, and PASP), showed no statistically significant differences between groups ( Table 4 ). These findings are consistent with the Cox proportional hazards model results presented in Table 5 and Table 6 , in which diabetes and atrial fibrillation emerged as independent predictors of embolic events.

**Table 4:** – Comparison between patients with and without thromboembolic events

Variable	Event (n = 14)	No event (n = 60)	p-value
Age, years	57.5 (44.2-62.0)	60.5 (52.8-67.0)	0.120
Female sex	11 (78.6%)	40 (66.7%)	0.520
White race	13 (92.9%)	48 (80.0%)	0.440
Diabetes mellitus	1 (7.1%)	23 (38.3%)	0.020
Hypertension	9 (64.3%)	47 (78.3%)	0.300
Pedunculated tumor	12 (85.7%)	37 (63.8%)	0.200
Obstructive pattern	8 (57.1%)	19 (31.7%)	0.120
Postoperative AF	5 (35.7%)	1 (1.6%)	0.002
Tumor length (mm)	42.5 (35.0-53.8)	45.0 (27.0-57.8)	0.910
Tumor width (mm)	32.5 (26.2-43.8)	35.0 (20.8-42.0)	0.530

Values are presented as n (%) for categorical variables and median [IQR] for continuous variables. P-values were calculated using Fisher’s exact test or the Mann–Whitney U test, as appropriate. AF: atrial fibrillation; IQR: interquartile range.

**Table 5:** – Univariate Cox proportional hazards regression for thromboembolic events

Variable	HR	95% CI	p-value
Age (per year)	0.96	0.92-1.00	0.06
Dyslipidemia	1.02	0.31-3.30	0.97
Hypertension	2.00	0.57-7.03	0.27
Preoperative reduced LVEF	2.22	0.62-7.86	0.21
LV diastolic diameter (dilated)	0.33	0.06-1.60	0.17
Increased PASP	1.08	0.47-2.49	0.84
Mobile myxoma	1.87	0.52-6.71	0.33
Tumor length (mm)	0.99	0.96-1.02	0.67
Tumor width (mm)	1.00	0.96-1.03	0.98

HR: hazard ratio; LV: left ventricle; LVEF: LV ejection function; PASP: pulmonary artery systolic pressure.

**Table 6:** – Multivariate Cox proportional hazards regression for thromboembolic events

Variable	HR	95% CI	p-value
Diabetes mellitus	0.11	0.006-0.740	< 0.05
Postoperative AF	6.74	1.49-30.51	< 0.05

AF: atrial fibrillation; HR: hazard ratio.

### Predictors of thromboembolic events

The multivariable Cox regression model ( Table 6 ) showed that LA dilation was associated with a lower, although non-significant, risk of thromboembolic events. Diabetes and atrial fibrillation were significantly associated with the hazard of thromboembolic events (HR = 0.11; 95% CI: 0.006-0.740; p < 0.05). Mobile tumors showed a trend toward a higher risk of thromboembolism, although this increase was not statistically significant.

Univariate or multivariate analyses of myxoma recurrence and mortality were not performed due to the small number of events. A focused analysis was conducted to explore variables associated with an increased risk of thromboembolic events. Univariate analysis identified diabetes as a potential protective factor, contrasting with its established role in increasing thromboembolic risk in the general population. This finding suggests that the myxoma itself may be the primary driver of thromboembolic risk in this population, supporting the rationale for early surgical removal.

## Discussion

This study reinforces the critical role of early surgical intervention in the management of cardiac myxomas and highlights its association with excellent long-term outcomes.^7^In this cohort of 74 patients, predominantly with sporadic tumors, the median follow-up was 60 months, during which survival reached 98.6% and tumor recurrence remained low at 2.7%. These findings are consistent with previous reports demonstrating the effectiveness of prompt resection in reducing mortality and recurrence, particularly when complete excision of the tumor and its attachment is achieved.^8 , 9^

The lower frequency of constitutional symptoms observed in our cohort contrasts with the higher prevalence reported in other series. This difference may be explained by referral patterns, as many patients were evaluated at our center at more advanced stages of disease, when obstructive and embolic manifestations may predominate over systemic symptoms.^10 - 12^

All procedures in our cohort were performed via median sternotomy, which remains the standard approach at our institution to ensure adequate exposure and complete tumor excision. Minimally invasive and robotic techniques, such as those using the DaVinci Xi^®^system, were not available during the study period. However, these approaches have been associated with shorter recovery times and improved postoperative quality of life in selected centers.^13 , 14^

The interatrial septum, particularly the fossa ovalis, is recognized as the most common site of origin for cardiac myxomas. En bloc resection with removal of a portion of the interatrial septum followed by patch reconstruction is often recommended to minimize recurrence. In our cohort, only 14.9% of patients underwent interatrial patch reconstruction. This relatively low rate reflects a tailored, real-world surgical strategy at our tertiary center, where operative decisions were guided by tumor size, attachment morphology, and intraoperative findings.^15 - 18^

In many cases, small pedunculated tumors allowed for complete excision with a narrow margin and primary septal closure, particularly in older or high-risk patients.^18 - 20^

The incidence of thromboembolic events in our cohort (18.9%), primarily ischemic stroke (13.5%), is comparable to rates reported in prior studies. Stefanou et al. (2018) described a stroke rate of 11% among patients with cardiac myxomas, while Pinede et al. (2001) reported thromboembolic events in approximately 30% of cases. Importantly, our multivariable Cox regression analysis did not identify tumor location or age as significant predictors of thromboembolic risk. Tumor mobility showed a trend toward increased risk, although this association did not reach statistical significance, likely due to the limited number of events. This observation is consistent with the meta-analysis by Liu et al. (2020), which highlighted the lack of consistent morphological predictors across studies.^3 , 21 , 22^

Postoperative atrial fibrillation emerged as an independent predictor of thromboembolic events in our cohort, with more than a sixfold increase in hazard. This finding reinforces the well-established association between new-onset atrial fibrillation and heightened embolic risk following cardiac surgery and underscores the importance of close rhythm monitoring and timely anticoagulation in this population.^23 , 24^

In the multivariable model, diabetes mellitus was associated with a lower hazard of thromboembolic events. This observation has not been consistently reported in previous studies and should be interpreted cautiously. It is likely exploratory and potentially confounded, rather than indicative of a true protective biological effect.^25 - 27^Possible explanations include closer clinical monitoring, more frequent use of antithrombotic or statin therapy, or unmeasured differences in cardiovascular risk management among diabetic patients. Because of the small number of events and the retrospective design, this finding should be considered hypothesis-generating and requires confirmation in larger prospective studies.

The cohort demonstrated a significant postoperative reduction in PASP (Wilcoxon Z = −2.070; p = 0.038), consistent with relief of LA inflow obstruction, particularly in patients with obstructive myxomas.

Conversely, LVEF declined in the early postoperative period (preserved LVEF 93.2% preoperatively vs 78.9% postoperatively; severe reduction 2.7% vs 15.5%; Wilcoxon Z = 3.052; p = 0.002). This pattern likely reflects transient perioperative ventricular dysfunction rather than a sustained deterioration in systolic function, especially considering that postoperative echocardiograms were performed within 30 days of surgery. These findings suggest that surgery promptly alleviates hemodynamic burden, while recovery of ventricular function may occur more gradually and warrants longitudinal echocardiographic follow-up.

Mikus et al. (2025) reported preserved LVEF in the postoperative period, indicating that the changes observed in our cohort may represent early, transient alterations rather than long-term impairment.^14^

Lastly, the survival rate observed in our study (98.6% over a median follow-up of 60 months) compares favorably with global surgical series, including the 93%-97% long-term survival reported by Jiang et al. (2019) and Perek et al. (2011). These results reinforce the long-term safety and effectiveness of surgical treatment for cardiac myxomas when performed in specialized centers.^7 , 9 , 28^

### Study limitations

The retrospective, single-center design and the relatively small sample size may limit the generalizability of these findings. Additionally, due to the long data collection period inherent to retrospective studies, certain potentially relevant variables, such as tumor morphology and surface characteristics, which may be associated with mobility and thromboembolic risk, were not consistently documented.

Furthermore, the observed postoperative reduction in LVEF may partly reflect perioperative myocardial injury or infarction, which was not systematically assessed or recorded in our cohort, limiting the interpretation of this finding.

## Conclusion

Our study reinforces that cardiac myxoma, when treated promptly, is associated with an excellent prognosis. At the same time, it highlights the significant thromboembolic risk related to this condition, which appears to be largely independent of tumor morphology. These findings support early surgical resection, even in asymptomatic patients and those with non-obstructive lesions. Continuous follow-up remains essential to monitor for late complications and potential recurrence.
